# Immunotherapy-Induced Complete Response in dMMR Rectal Cancer—A Surgical Dilemma?

**DOI:** 10.3390/cancers17193153

**Published:** 2025-09-28

**Authors:** Panagiotis Loufopoulos, Konstantinos Perivoliotis, Danai Chatziathanasiou, Maximos Frountzas, Anisha Sukha, Abdullah Alrebdi, Mohammad Mahmoud Rajab Eddama, Christos Kontovounisios, Shengyang Qiu, Paris Tekkis, Shahnawaz Rasheed

**Affiliations:** 1 Department of Colorectal Surgery, Royal Marsden Hospital, NHS Foundation Trust, London SW3 6JJ, UK; konstantinos.perivoliotis@rmh.nhs.uk (K.P.);; 2 Department of Breast Surgery, Royal Marsden Hospital, NHS Foundation Trust, London SW3 6JJ, UK; 3 Research Department of Surgical Biotechnology, Division of Surgery and Interventional Science, University College London, London W1W 7TY, UK; 4 Department of Colorectal Surgery, Chelsea and Westminster Hospital, NHS Foundation Trust, London SW3 6JJ, UK; 5 Department of Surgery and Cancer, Imperial College London, London W12 0BZ, UK

**Keywords:** dMMR, rectal cancer, neoadjuvant immunotherapy, complete response, surgical dilemma, organ preservation, watch-and-wait

## Abstract

**Simple Summary:**

Deficient mismatch repair rectal cancer represents a distinct molecular subtype comprising approximately 10% of rectal malignancies. These tumours exhibit remarkable susceptibility to immune checkpoint inhibitors, with clinical studies reporting complete tumour regression rates approaching 100% following neoadjuvant immunotherapy. This therapeutic breakthrough has created a significant clinical dilemma regarding optimal patient management: whether individuals achieving complete response should proceed with standard surgical resection or pursue organ preservation through intensive surveillance protocols. This comprehensive review evaluates the comparative evidence between watch-and-wait management strategies and conventional surgical approaches in complete responders. Current evidence demonstrates equivalent oncological outcomes between both treatment paradigms, with organ preservation approaches maintaining normal gastrointestinal, genitourinary, and sexual function. This breakthrough is particularly important for younger patients who may live for decades, as it offers the possibility of curing cancer while preserving normal body function and quality of life.

**Abstract:**

Background: Deficient mismatch repair rectal cancer represents approximately 10% of rectal malignancies and demonstrates exceptional responsiveness to immune checkpoint inhibitors, achieving unprecedented complete response rates approaching 100%. This creates a novel clinical dilemma: should patients achieving complete response undergo standard surgical resection or pursue organ preservation through watch-and-wait management? Methods: We conducted a comprehensive literature review of clinical trials and retrospective studies published through 2025, focusing on response assessment strategies, decision-making frameworks, oncological outcomes, and quality of life assessments. Results: Landmark studies demonstrated remarkable efficacy with dostarlimab achieving 100% clinical complete response, while surgical cohorts achieved 68–92% pathological complete response rates. Response assessment challenges included pseudoprogression and pseudoresidue phenomena that complicated conventional imaging interpretation and required specialised multimodal evaluation protocols. Comparative analyses suggest equivalent oncological outcomes between surgical and non-surgical approaches in complete responders, achieving 100% disease-free survival at 2–3 years across multiple studies. The watch-and-wait approach offered significant advantages by preserving organ integrity and avoiding surgical morbidity, including permanent colostomy (15.4%) and perioperative complications (19.3%). Conversely, surgical management provided distinct benefits through definitive tissue confirmation and anxiety relief from intensive surveillance requirements and potential recurrence concerns. Conclusions: The surgery versus watch-and-wait dilemma represents a choice between equally effective oncological approaches with different quality of life implications. Evidence supports individualised decision-making weighing functional preservation benefits against patient preferences and institutional capabilities in this evolving therapeutic landscape.

## 1. Introduction

Rectal cancer management has traditionally centred on neoadjuvant chemoradiotherapy followed by total mesorectal excision (TME) for locally advanced disease [1]. However, the emergence of precision oncology has revealed distinct molecular subtypes that respond differently to conventional treatments, fundamentally challenging the “one-size-fits-all” approach.

Deficient mismatch repair (dMMR) tumours, characterised by microsatellite instability-high (MSI-H) status, represent a unique molecular subset comprising approximately 10% of rectal malignancies [2]. These tumours harbour exceptional biological characteristics with high mutational burden, extensive neoantigen load, and dense CD8+ T-cell infiltration, creating an intensely immunogenic phenotype highly susceptible to immune checkpoint inhibition [3,4]. The majority of dMMR rectal cancers (~84%) are associated with Lynch syndrome (hereditary nonpolyposis colorectal cancer), with germline mutations in the mismatch repair genes MLH1, MSH2, MSH6, PMS2 or EPCAM. This contrasts with proximal colon cancers, where sporadic MLH1 promoter hypermethylation represents a more common mechanism of mismatch repair deficiency [2,5].

The therapeutic landscape was revolutionised by landmark trials demonstrating remarkable immunotherapy efficacy in dMMR colorectal cancer. The KEYNOTE-016 study established that mismatch repair deficiency predicts exceptional response to PD-1 blockade [6], while CheckMate-142 demonstrated objective response (OR) rates of 69% with combination immunotherapy [7]. The KEYNOTE-177 trial validated pembrolizumab as first-line therapy for metastatic colorectal cancer, achieving superior outcomes compared to chemotherapy [8]. These findings led to FDA approval of immune checkpoint inhibitors (ICI) for MSI-H/dMMR solid tumours, marking the first tissue-agnostic cancer therapy approval [9].

The transition to neoadjuvant immunotherapy (nIT) has yielded unprecedented results. The NICHE trials demonstrated 68–69% pathological complete response (pCR) rates [10,11,12], while the dostarlimab study by Cercek et al. achieved 100% clinical complete response (cCR) [13]. This contrasts with conventional chemoradiotherapy, to which dMMR rectal tumours appear to exhibit resistance [14].

These exceptional outcomes have created a novel clinical dilemma: should patients achieving complete response (CR) undergo standard surgical resection, or can surgery be safely omitted? This question assumes particular urgency in rectal cancer, where surgery carries significant morbidity including permanent colostomy, urinary and sexual dysfunction, and anastomotic complications. The younger age typical of dMMR rectal cancer, often associated with Lynch syndrome, amplifies the importance of quality of life preservation over decades of life expectancy [15].

The aim of this comprehensive review is to synthesise evolving evidence regarding immunotherapy-induced complete responses in dMMR rectal cancer, to examine surgical decision-making challenges, and to provide an evidence-based framework for navigating this paradigm-shifting therapeutic landscape. The goal is to optimise outcomes by balancing unprecedented organ preservation opportunities with oncological safety and patient-centred care in this rapidly evolving field.

## 2. Clinical Evidence for Immunotherapy in dMMR Colorectal Cancer

### 2.1. Definitions of Complete Response

Clinical complete response (cCR) was defined as the absence of detectable tumour on digital rectal examination, endoscopy, and cross-sectional imaging, with negative biopsy when performed. Pathological complete response (pCR) was defined as the absence of viable tumour cells in the resected specimen (ypT0N0) on histopathological examination. Disease-free survival (DFS) was defined as the time from the date of treatment initiation to the first documented local or distant recurrence or death from any cause, whichever occurred first. These definitions are applied consistently throughout this review.

### 2.2. Landmark Clinical Trials

#### 2.2.1. KEYNOTE-177: Foundation for Immunotherapy

The KEYNOTE-177 trial (Lancet Oncol 2022) established pembrolizumab as first-line therapy for MSI-H/dMMR metastatic colorectal cancer and provided the foundational evidence for immunotherapy efficacy in this population. This randomised phase III study demonstrated superior efficacy compared to standard chemotherapy across multiple endpoints. The objective response (OR) rate was 43.8% with pembrolizumab versus 33.1% with chemotherapy, while radiological complete response (rCR) rates were 13.1% versus 3.9%, respectively. Median progression-free survival (PFS) favoured pembrolizumab at 16.5 months compared to 8.2 months with chemotherapy, and the treatment demonstrated superior tolerability with lower grade 3–4 adverse events (22% versus 66%) [8] (Table 1). All reported efficacy and safety outcomes in this study are derived from its original publication.

#### 2.2.2. CheckMate-142 & -8HW: Combination Immunotherapy

The CheckMate-142 study (JCO 2022) evaluated nivolumab with or without ipilimumab in dMMR metastatic colorectal cancer and provided important insights into combination immunotherapy approaches. Nivolumab monotherapy achieved a 31.1% OR rate, while the combination of nivolumab plus ipilimumab demonstrated a 69% OR rate with a 13% CR rate. Durable responses were maintained after a median 29 months of follow-up, though toxicity increased with combination therapy (Table 1). The study established that combination immunotherapy could achieve higher response rates at the cost of increased toxicity, informing subsequent neoadjuvant study designs [7].

The CheckMate-8HW study (NEJM 2024) compared the efficacy between nivolumab plus ipilimumab and chemotherapy in patients with metastatic colorectal cancer who had not previously received systemic treatment. A total of 303 patients were randomly assigned to receive nivolumab plus ipilimumab or chemotherapy. 255 patients had centrally confirmed MSI-H or dMMR tumours. According to the results, the 24-month PFS was 72% with nivolumab plus ipilimumab compared to 14% with chemotherapy. In addition, the restricted mean survival time was longer, and grade 3 or 4 adverse events were fewer in the nivolumab plus ipilimumab group comparing to chemotherapy group [25] (Table 1). All reported efficacy and safety outcomes for the CheckMate studies discussed in this section are derived from their respective original publications.

#### 2.2.3. NICHE Studies: Neoadjuvant Immunotherapy

The NICHE trials pioneered nIT in dMMR colon cancer and provided proof-of-concept for this approach. NICHE-1 was a single-arm study of 32 patients with non-metastatic resectable dMMR colon cancer treated with ipilimumab plus nivolumab. The study achieved 69% pCR in dMMR tumours, with minimal surgical delay and acceptable toxicity profile. The median follow-up was around 25 months, with no disease recurrences reported during that period [10] (Table 1).

NICHE-2 expanded the cohort to 111 patients and validated the initial findings with a 68% pCR rate. Grade 3-4 immune-related adverse events occurred in only 4% of patients, and no disease recurrence was observed at 26 months median follow-up. The 3-year disease free survival (DFS) was 100% (Table 1). These studies established the feasibility and efficacy of nIT in dMMR colon cancer [11].

In the most recent NICHE-3 study, 59 patients were treated with a short regimen of two cycles of nivolumab plus relatlimab on day 1 and day 29, followed by surgery 6-8 weeks after enrolment. The pCR rate was 68% and the grade 3–4 adverse events were 10%. After a median follow-up of 8 months, all patients were alive, and 98% of patients were disease free [12] (Table 1).

#### 2.2.4. Dostarlimab: The 100% Complete Response Study

Perhaps the most striking results emerged from the single-institution dostarlimab study conducted at Memorial Sloan Kettering (MSK) Cancer Centre. This single-arm phase II study enrolled 12 patients with stage II-III dMMR rectal cancer and treated them with dostarlimab 500 mg every 3 weeks for 6 months. The primary endpoint was sustained cCR at 12 months. Remarkably, 100% cCR was achieved in all 12 enrolled patients who have undergone at least 6 months of follow-up (median follow-up time was 12 months) (Table 1). No patient required chemoradiotherapy or surgery during the follow-up period, representing the first report of 100% CR rate in solid tumour nIT. However, the primary end point was not reported in its finality. This study garnered significant attention and highlighted the exceptional responsiveness of dMMR rectal cancer to immunotherapy. Nevertheless, the small sample size and short follow-up period necessitated validation in larger cohorts with extended observation periods [13].

A more recent phase II study, published by the same authors in 2024, included 41 patients who completed the full course treatment (6 months of dostarlimab), and all of them demonstrated a cCR. None required any supplementary treatments, and neither recurrences nor metastasis were observed. A total of 20 patients met the criteria for sustained CR during a median observation period of 28.9 months from treatment initiation, successfully meeting the primary endpoint which was not reported in the previous study [16] (Table 1).

### 2.3. Additional Phase II Clinical Trials

#### 2.3.1. PICC Study

This randomised phase II trial included 34 patients with locally advanced dMMR colorectal cancer, and compared the efficacy between toripalimab plus celecoxib versus toripalimab alone. The results demonstrated 88% pCR in the combination therapy group, compared to 65% in the monotherapy group. Only 3% of patients receiving combination therapy had 3–4 adverse events, and the median follow-up time was 14.9 months. No recurrences were reported, but longer term follow-up are needed to assess the effects on survival-related endpoints [17] (Table 1).

#### 2.3.2. Pembrolizumab Neoadjuvant Study

This Danish multicentre, investigator-initiated, single-arm phase II study evaluated the safety and efficacy of neoadjuvant pembrolizumab in 42 patients with stage I–III dMMR colon cancer. All patients received pembrolizumab and underwent surgery, except one patient who refused surgery. Patients received a single 4 mg/kg dose of pembrolizumab followed by surgery 3–5 weeks later (median 32 days). A pre-planned interim analysis assessed safety and efficacy, with pCR > 20% as the primary efficacy endpoint. Treatment was generally well tolerated. Surgical complications occurred in 16 of 41 patients (39%), with three above Clavien–Dindo grade 2, including two grade 3b events and one surgery-related grade 5 gastric ulcer perforation. Three grade 3 adverse events were reported, with no grade 4 events. Efficacy outcomes were encouraging: 46% of evaluable patients achieved a pCR, and 61% demonstrated a major pathological response, exceeding the predefined threshold for efficacy [18] (Table 1).

#### 2.3.3. Sintilimab Neoadjuvant Study

A total of 16 patients with dMMR locally advanced rectal cancer were included in this single-arm phase II study, and they were treated with neoadjuvant sintilimab monotherapy. Among these patients, 6 underwent surgery, of whom 3 had pCR; and 9 patients had cCR and followed the watch-and-wait (WW) strategy. Only 1 patient had a serious adverse event and discontinued therapy. A CR rate of 75% was noted, and after a median follow-up of 17.2 months all patients were alive and none had disease recurrence. Only a 6% of the patients had grade 3–4 adverse events (Table 1). The study suggested that anti-PD-1 monotherapy is effective and tolerable for patients with dMMR locally advanced rectal cancer and could potentially spare some patients from radical surgery [19].

### 2.4. Retrospective Studies and Case Series

#### 2.4.1. A Single-Centre Real-World Study

This study was conducted at Yunnan Cancer Hospital and included 32 patients with dMMR locally advanced colorectal cancer (8/32 rectal cancer). All the patients received nIT with a single-agent PD-1 inhibitor. Among them, 3 patients with locally advanced rectal cancer achieved cCR and followed the WW strategy. The rest 29 patients were submitted to radical surgery with a pCR of 75.9%. The median follow-up time was 14 months, during which no local recurrence or distant metastasis was found in all patients. No grade 3–4 adverse events were reported [20] (Table 1).

#### 2.4.2. A Multiple-Centre Cohort Study

Patients with locally advanced dMMR rectal cancer that received nIT were included in that study. The PD-1 inhibitors used included pembrolizumab, sintilimab, and tislelizumab (monotherapy). A total of 20 patients were included in the study, out of which 13 patients underwent TME after immunotherapy and 7 patients followed the WW approach. The pCR rate among those that underwent surgery was 84.6%. The median follow-up period was 25 months, during which no local recurrences or distant metastasis were observed. The 2-year disease survival was 100% in all patients. No grade 3–4 adverse events were reported (Table 1). The study found that 100% (7/7) of patients who were managed with a WW strategy had a comparable oncological safety profile as those who accepted TME surgery. More importantly, patients in the WW group did not experience a reduced quality of life associated with surgical complications, enterostomies, or deterioration of the bowel, urinary system, or sexual function [21].

#### 2.4.3. Long Term Outcomes Study

A total of 24 patients who achieved cCR after nIT for locally advanced dMMR rectal cancer were included in the study. They were not submitted to surgery and followed the WW approach. The median follow-up period after the cCR was 29.1 months, during which no local regrowth or distant metastasis were observed. The grade 3–4 adverse events were 8.4%. The 3-year disease-free and overall survivals were both 100% (Table 1). These results indicated that the watch-and-wait strategy for cCR after nIT for dMMR rectal cancer had promising long-term outcomes [22].

#### 2.4.4. Chinese Multicentre Experience

A retrospective analysis of 29 dMMR rectal cancer patients treated with anti-PD-1 immunotherapy across six institutions in China demonstrated consistent efficacy across different geographic and healthcare settings. Treatment approaches included pembrolizumab, toripalimab, sintilimab, camrelizumab, or nivolumab as monotherapy or in combination with other agents. Among these patients, 20 of them achieved cCR (69%), and out of those, 19 approached the WW strategy. The rest 10 patients underwent surgery, of whom 5 demonstrated pCR. In total, the CR rate (clinical plus pathological) was 82.9%. The grade 3–4 adverse event rate was 15%. During a median follow-up of 17.1 months, no local or distant recurrence was observed, resulting in 100% 2-year local recurrence-free, distant metastasis-free, disease-free, and overall survival rates [23] (Table 1).

#### 2.4.5. Sun Yat-Sen University Series

A comprehensive analysis of 13 MSI-H/dMMR rectal cancer patients receiving neoadjuvant ICI revealed important insights into response patterns and assessment challenges. Among these patients, 11 underwent TME, 1 transanal local excision, and 1 followed the WW approach. The study achieved approximately 92% pCR, but identified unique response patterns including 23.1% pseudoprogression and 76.9% pseudoresidue (Table 1). Histopathological examination revealed distinctive immune-related regression features including dense lymphocyte infiltration, fibrogenesis, and plasma cell infiltration. These findings highlighted the inadequacy of standard radiological assessment for immunotherapy response evaluation [24].

## 3. The Surgical Dilemma: Response Assessment and Decision-Making

### 3.1. Challenges in Response Evaluation

#### 3.1.1. Pseudoprogression and Pseudoresidue

ICI in dMMR rectal cancer produce distinctive response patterns that fundamentally differ from conventional therapy responses. In the study conducted by Xie et al., 13 patients with locally advanced dMMR rectal cancer receiving nICIs were evaluated. Tumour response was assessed by thoraco-abdominopelvic CT and pelvic MRI at baseline, 7–13 days after the first half of scheduled cycles, and before surgery, with at least three imaging assessments per patient. Colonoscopy and endorectal ultrasound were performed at baseline and optionally at subsequent timepoints [24].

Treatment response was initially evaluated according to RECIST 1.1—where progressive disease (PD) was defined as a ≥20% increase in the sum of target lesion diameters (with an absolute increase ≥5 mm) or the appearance of new lesions—and subsequently reassessed using iRECIST criteria. Under iRECIST, such findings were first classified as unconfirmed PD (iUPD) and required confirmation at the next assessment; if progression was verified, it was designated as confirmed PD (iCPD) [24].

Pathologic evaluation of surgically resected specimens was performed according to AJCC staging, with tumour regression graded using the AJCC TRG system by two board-certified pathologists. Patients with no residual tumour were considered to have achieved pCR. Histologic features of regression and immune activation—including lymphocytic infiltration, fibrosis, and necrosis—were assessed, and residual viable tumour percentage was calculated [24].

Using these assessments, pseudoprogression occurred in 23.1% of patients, representing transient radiologic progression due to immune cell infiltration rather than true tumour growth. Pseudoresidue was observed in 76.9% of patients, reflecting persistent radiologic abnormalities from immune-mediated tissue remodelling despite complete eradication of viable tumour cells [24].

#### 3.1.2. Limitations of Conventional Imaging

Standard imaging modalities demonstrate particular limitations when applied to immunotherapy-treated dMMR rectal cancer. The intense inflammatory response characteristic of effective immunotherapy creates persistent radiological abnormalities that confound conventional interpretation. According to studies, magnetic resonance tumour regression grade (mrTRG), while excellent for chemoradiotherapy response assessment, struggles to distinguish immune-mediated fibrosis from residual tumour in immunotherapy-treated patients. In addition, the inflammatory microenvironment generated by successful immunotherapy in dMMR rectal cancer creates persistent fluorodeoxyglucose (FDG) uptake on positron emission tomography (PET), leading to false positive interpretations in patients who have achieved pCR [26]. Early post-treatment scans (within 2–4 weeks) show poor specificity (50–60%) because of persistent inflammatory uptake, whereas delaying imaging to beyond 8 weeks improves specificity as inflammation subsides. These findings, however, are derived primarily from studies in the context of neoadjuvant chemoradiotherapy; for patients receiving neoadjuvant immunotherapy, evidence on optimal timing and false-positive rates is not yet available [27]. This represents a fundamental limitation of metabolic imaging in the immunotherapy era, where inflammatory activity may persist for months after complete tumour eradication.

### 3.2. Response Assessment Strategies

#### 3.2.1. Endoscopic Evaluation

The endoscopic appearance of immunotherapy-induced CR often reveals distinctive features including mucosal healing, absence of visible tumour mass, and characteristic fibrotic changes, with the limitation of assessing only luminal regression and no extraluminal high risk features. Fox et al. demonstrated that endoscopic assessment provides valuable correlation with pathological response status, though interpretation requires familiarity with the unique appearance of immune-mediated tumour regression [28]. Biopsy sampling from the original tumour bed allows histological confirmation of response status, revealing the characteristic histological features of immunotherapy-induced regression including dense lymphocytic infiltration, fibrosis, and absence of viable tumour cells [24].

#### 3.2.2. Biomarker Integration

Circulating tumour DNA (ctDNA) has emerged as a particularly valuable tool for monitoring immunotherapy response in dMMR rectal cancer. The clearance kinetics of ctDNA may provide early indicators of treatment response, with negative ctDNA levels correlating strongly with sustained CR, as has also been observed with standard chemoradiotherapy. This molecular approach offers particular advantages in immunotherapy-treated patients where conventional imaging may be compromised by inflammatory changes. Traditional tumour markers including CEA and CA 19–9 demonstrate dynamic clearance patterns that correlate with immunotherapy response quality [29,30].

#### 3.2.3. Imaging Techniques

Functional MRI sequences, including diffusion-weighted imaging (DWI) and dynamic contrast enhancement (DCE), offer improved discrimination between immune-mediated fibrosis and residual tumour in dMMR rectal cancer patients. The apparent diffusion coefficient (ADC) measurements from DWI-MRI may distinguish the high cellularity of residual tumour from the low cellularity characteristic of immune-mediated fibrosis [31]. Quantitative DWI and DCE-MRI metrics offer potential as adjuncts for response assessment in ICI-treated dMMR rectal cancer, but their interpretation remains challenging. Mean apparent diffusion coefficient (ADC) values for rectal tumours typically range from 1.2 to 1.4 × 10^−3^ mm^2^/s and have demonstrated high repeatability, with a coefficient of variation (CoV) around 17%. In contrast, DCE-MRI parameters [(volume transfer constant (K^trans^), plasma volume fraction (V_p_), revere volume transfer constant (K_ep_)] exhibit greater variability due to tumour heterogeneity and differences in acquisition protocols and pharmacokinetic modelling. Notably, no validated cutoffs currently exist to distinguish pseudoprogression from true progression or pseudoresidue in this setting, highlighting the need for prospective studies with pre-specified thresholds, test–retest validation, and cross-scanner reproducibility assessments [32,33]. MRI can be used in combination with computed tomography and positron emission tomography (PET) to restage the tumour following immunotherapy [31].

### 3.3. Decision-Making Framework

#### 3.3.1. cCR Criteria–Clinical Decision Factors

**Figure 1:**
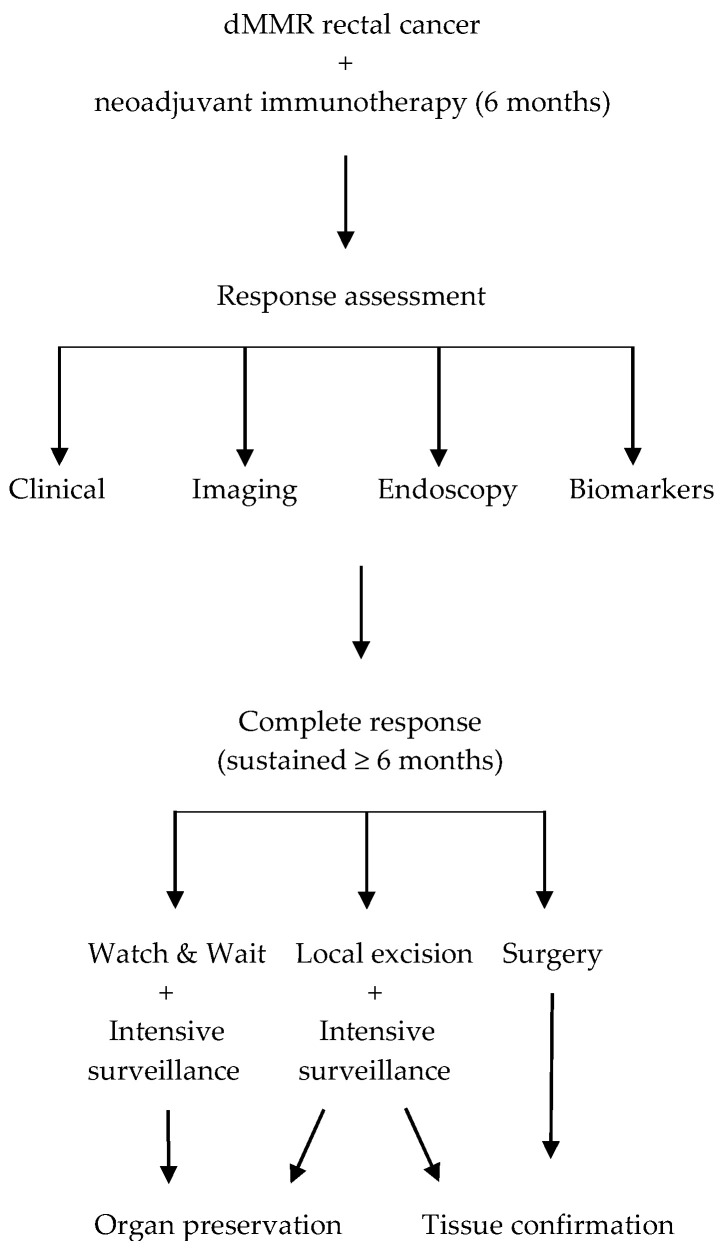
Treatment decision algorithm.

Essential requirements include absence of visible or palpable tumour on clinical examination, negative biopsies from the tumour bed demonstrating absence of viable tumour cells, normalised biomarkers including ctDNA, CEA and CA 19–9, and stable imaging without evidence of disease progression. The decision between surgery and organ preservation following immunotherapy-induced CR requires comprehensive assessment of multiple factors. Clinical factors include the quality and durability of response, with sustained complete response for at least 6 months—defined as the complete disappearance of the tumour, confirmed by imaging, clinical examination, endoscopy, and biomarkers, with no evidence of recurrence or progression—generally favouring organ preservation approaches [13,16] (Figure 1). Tumour factors including stage, location, and accessibility for ongoing surveillance affect treatment recommendations. Patient factors encompass age, comorbidities, functional status, compliance ability, and personal preferences regarding treatment approach. Institutional factors include surgical expertise, immunotherapy experience, and multidisciplinary team capabilities [34].

#### 3.3.2. Treatment Selection Paradigms

Based on current evidence, neoadjuvant immunotherapy is recommended for dMMR rectal tumours by leading guidelines: NCCN Guidelines Version 3.2025 (category 2A recommendation), ESMO Clinical Practice Guideline 2025 (level IIA evidence), and ASCO Guideline (low-quality evidence; strong recommendation) [35,36].

The immunotherapy-first approach employs neoadjuvant immunotherapy for 6 months followed by comprehensive response assessment using multimodal evaluation and treatment decisions based on response status.

For patients achieving complete response to immunotherapy, three primary options can be considered:

Watch-and-wait approach with intensive surveillance provides organ preservation with careful monitoring, building on the established safety of this approach while incorporating the enhanced response rates observed with immunotherapy [13,16,19,20,21,22,23,24] (Figure 1);
Local excision offers tissue confirmation while preserving organ function, providing pathological verification of complete response while minimising surgical morbidity (Figure 1). Favourable candidates for local excision are those with tumours located within 8–10 cm of the anal verge, measuring <4 cm in size, and readily accessible for full-thickness endoscopic excision. Additional selection criteria include a marked response to therapy and the absence of high-risk imaging features such as threatened mesorectal fascia, extramural vascular invasion, or bulky nodal involvement [24,37,38];
Standard surgery remains an option based on patient preference (Figure 1), though the exceptional response rates observed with immunotherapy in dMMR rectal cancer challenge the routine application of radical surgery in complete responders [19,20,21,23,24].

## 4. The Watch-and-Wait Approach

### 4.1. Immunotherapy-Specific Considerations for Organ Preservation

#### 4.1.1. Enhanced Complete Response

The landmark dostarlimab study by Cercek et al. achieved 100% cCR in 41 patients, with all patients maintaining sustained responses at median follow-up of 28.9 months [13,16]. Similarly, the NICHE studies demonstrated 68–69% pathological complete response rates with combination immunotherapy approaches [10,11,12]. The durability of responses appears exceptional, with sustained responses at 2+ years in early studies suggesting potential long-term immunity. Yu et al. reported 100% DFS at 29.1 months median follow-up in 24 patients managed with WW following immunotherapy-induced CR [26].

#### 4.1.2. Different Response Kinetics and Immune Memory

Immunotherapy response patterns differ fundamentally from chemoradiotherapy in several important ways. Delayed responses may continue improving months after treatment completion, creating uncertainty about optimal timing for response assessment [13,16]. The potential for immune memory may provide long-term immunity against tumour antigens, distinguishing immunotherapy responses from conventional treatment effects and potentially offering superior protection against recurrence [3]. Pseudoprogression patterns can create initial apparent worsening before improvement, according to Xie et al., requiring careful clinical judgement to avoid premature intervention. The extensive pseudoresidue creates unique monitoring challenges, but does not compromise the safety or efficacy of organ preservation approaches. The management of pseudoprogression and pseudoresidue relies on multimodal evaluation to avoid misclassification. Declining circulating tumour DNA (ctDNA) levels can indicate true response, while endoscopic or ultrasound-guided biopsies provide direct pathological confirmation. As most pseudoprogression occurs within the first three months of ICI therapy, extending the observation period combined with these modalities improves the accuracy of response assessment [24].

### 4.2. Patient Selection Criteria

Tumour Characteristics:

- Confirmed dMMR/MSI-H status through validated testing methods;
- Local disease without distant metastases;
- Accessibility for surveillance procedures (Table 2).

Response Characteristics:

- Clinical complete response by multimodal assessment;
- Sustained response for at least 6 months;
- Negative ctDNA when available (Table 2).

Patient Factors:

- Thorough understanding of the immunotherapy approach;
- Ability to comply with intensive follow-up requirements;
- Strong preference for organ preservation (Table 2).

**Table 2:** Summary of factors favouring Watch-and-Wait versus Surgery.

Watch-and-Wait	Surgery
Sustained complete response ≥6 months	Incomplete or partial response
Young age with quality of life priorities	Patient preference for definitive treatment
Strong patient preference for organ preservation	Compliance concerns with intensive surveillance
Excellent compliance potential	Limited access to specialised monitoring
Access to specialised surveillance	High-risk tumour features

### 4.3. Surveillance Protocols

Intensive surveillance represents a crucial component of safe WW implementation following immunotherapy, requiring carefully designed protocols that balance early detection of recurrence with patient quality of life, while accounting for the unique response patterns of ICI (Table 3).

### 4.4. Salvage Surgery Considerations

Salvage surgery criteria must be clearly defined to ensure prompt intervention when indicated. Local regrowth indicators include visible or palpable tumour recurrence, progressive imaging changes not attributable to inflammatory response, rising biomarkers suggesting disease recurrence, and positive surveillance biopsies demonstrating viable tumour cells. Kong et al. reported that salvage surgery was possible in 83.8% out of 256 patients with persistent cCR after neoadjuvant chemoradiotherapy [39]. While studies have validated the success of salvage surgery after neoadjuvant chemoradiotherapy in rectal cancer, evidence remains insufficient to support this approach in the immunotherapy setting.

### 4.5. Clinical Outcomes

#### 4.5.1. Exceptional Efficacy Results

Cercek A. et al. reported that 20 patients with sustained cCR had no recurrences or metastasis after a median follow-up of 28.9 months [16]. Yu et al. reported 100% DFS and overall survival at 29.1 months median follow-up in 24 patients managed with WW [22]. Yang et al. demonstrated 100% DFS at 25 months median follow-up in 7 patients managed with WW, with comparable outcomes to patients undergoing surgery [21]. The Chinese multicentre experience, reported by Wang et al., included 19 patients managed with WW following immunotherapy-induced CR with 100% 2-year local recurrence-free, distant metastasis-free, disease-free, and overall survival rates [23]. According to Chen G. et al., 9 patients with cCR followed the WW strategy and none of them had recurrence or distant metastasis after a median follow-up of 17.2 months [19]. Similarly, Zhang X. et al. reported no recurrence or distant metastasis after a median follow-up of 14 months in 3 patients that chose the WW approach [20].

#### 4.5.2. Functional Results

Yang et al. demonstrated that patients managed with WW did not experience a reduced quality of life associated with surgical complications, enterostomies, or deterioration of the bowel, urinary system, or sexual function [21]. The MSK experience showed patients have preserved normal bowel function, bladder function, sexual function, fertility following successful immunotherapy [13,16]. These benefits assume particular importance in the typically younger dMMR population, where decades of preserved function may be achieved. The Lynch syndrome association in 90% of cases means many patients are in reproductive years, making fertility preservation critical [15]. The median age of colorectal cancer onset in patients with Lynch syndrome, who carry proven germline MMR mutations, is approximately 45 years. In one study, total fertility among women was reduced by about 40% following a CRC diagnosis, with the greatest decline observed in younger patients (ages 20–24); the study population was followed to a median age of 55 years [40].

The absence of surgical morbidity including permanent colostomy risk, anastomotic complications, as well as anal sphincter, urinary and sexual dysfunction provides significant advantages. Patients with organ preservation have similar DFS and OS compared to those who undergo surgery, but with significantly improved quality of life [41]. However, beyond the potential benefits of organ preservation, ICI therapy carries important risks. Endocrine immune-related adverse events are relatively common, particularly thyroid dysfunction such as hypothyroidism (≈5–13%), while hypophysitis is less frequent (~3%) but occurs more often with combination regimens [42,43]. As these toxicities are often permanent, they may have significant implications for fertility and pregnancy, underscoring the need for proactive fertility counselling and consideration of preservation strategies in this patient population.

## 5. The Surgical Approach

### 5.1. Factors Favouring Surgery

#### 5.1.1. Patient Preference and Psychological Considerations

Despite achieving cCR to immunotherapy, some patients prefer definitive surgical treatment for psychological reassurance and pathological confirmation of response status. The investigational nature of nIT and limited long-term follow-up data may influence patient decision-making toward more established treatment approaches [9]. Yang et al. reported that among 20 dMMR rectal cancer patients achieving complete or near-complete response to immunotherapy, 13 patients chose surgery, while 7 elected WW management [21]. The younger age demographic typical of dMMR rectal cancer patients may paradoxically favour surgery in some cases, with decades of life expectancy making surgical cure attractive despite the associated morbidity. Patient anxiety regarding surveillance requirements and the potential for recurrence may influence treatment decisions toward definitive surgical management [44] (Table 2).

#### 5.1.2. Compliance and Access Considerations

The intensive surveillance requirements for WW management may not be feasible for all patients achieving CR to immunotherapy. Geographic barriers to specialised care, insurance coverage limitations, and inability to comply with frequent monitoring schedules may favour surgical approaches. Wang et al. noted that among 29 dMMR rectal cancer patients in their multicentre cohort, 10 patients underwent surgery despite good responses to immunotherapy, reflecting various patient and institutional factors influencing treatment decisions. Limited access to specialised centres with expertise in immunotherapy response monitoring and salvage surgery capabilities may influence treatment recommendations toward upfront surgical management. The requirement for multidisciplinary teams experienced in managing immunotherapy-treated patients may not be universally available, affecting treatment feasibility in some healthcare settings [23] (Table 2).

### 5.2. Surgical Outcomes

#### 5.2.1. Pathological Complete Response Confirmation

Surgery following immunotherapy-induced cCR frequently confirms exceptional pathological response rates, validating the efficacy of ICI in dMMR rectal cancer. The NICHE studies demonstrated pCR of 68–69% in dMMR colorectal cancer patients undergoing surgery after combination immunotherapy [10,11,12]. Zhang et al. reported 75.9% pCR in 29 patients with dMMR locally advanced colorectal cancer who underwent surgery following nIT [20]. Yang et al. documented 84.6% pCR among 13 dMMR rectal cancer patients who chose surgery after immunotherapy [21]. The Chen et al. study reported pCR in 3 of 6 dMMR rectal cancer patients who underwent surgery following sintilimab treatment, representing a 50% pCR rate in this surgical subset [19].

#### 5.2.2. Perioperative Safety and Complications

The timing and procedure of the operation are determined by the consultant surgeon after a comprehensive evaluation of the patient’s response to treatment and general condition. As reported in a study, the median time from the last nIT to surgery was 25 days [21]. The safety profile of surgery following immunotherapy appears comparable to conventional approaches, with no significant increase in perioperative complications or surgical morbidity. Cui et al. conducted a systematic review and meta-analysis demonstrating an overall complication rate of 19.3% following surgery after neoadjuvant immunotherapy for dMMR colorectal cancer, predominantly infection and anastomotic leakage (grade I-II) [45]. Chalabi et al.’s study reported an incidence of surgery-related adverse events of 19%, out of which grade III accounted for 10% and anastomotic leak was observed in 3% [46]. According to the multiple-centre cohort study performed by Yang R. et al., the surgery-related adverse events rate was 23.1%. Complications included a grade 1 surgical incision with poor healing, a grade 1 postoperative anastomotic bleeding, and a grade 2 anastomotic leak. None of the patients experienced perioperative mortality or severe surgery-related morbidity requiring re-operation [21].

### 5.3. Future Directions in Surgical Management

The development of minimally invasive surgical approaches may provide intermediate options between radical surgery and complete organ preservation. Local excision techniques offer pathological confirmation while preserving organ function, potentially providing optimal outcomes for selected patients [24]. These approaches require validation in immunotherapy-treated populations, but offer promising directions for personalised treatment strategies.

The integration of biomarker assessment including ctDNA monitoring may provide objective guidance for surgical decision-making. Patients with persistently negative ctDNA following immunotherapy may be optimal candidates for organ preservation, while those with detectable ctDNA might benefit from surgical intervention [29,30].

## 6. Comparative Outcomes: Watch-and-Wait Versus Surgery

### 6.1. Oncological Outcomes

Comparative studies suggest equivalent oncological outcomes between surgical and WW approaches in patients achieving CR to immunotherapy. Cercek A. et al. documented no recurrences or distant metastasis after a median follow-up of 28.9 months [16]. Yang et al. reported 100% DFS at 25 months median follow-up in both surgical and watch-and-wait groups, with no recurrences or metastasis [21]. Wang et al. documented 100% 2-year local recurrence-free, distant metastasis-free, disease-free, and overall survival rates across their entire cohort, encompassing both surgical and non-surgical management approaches [23]. Zhang et al. reported no recurrences or distant metastasis in both surgical and watch-and-wait groups after a median follow-up of 14 months [20]. Similarly, no recurrences or metastasis were observed in both groups after a median follow-up of 17.2 months, according to Chen G. et al. [19]. Yu et al. included 24 patients that followed the WW approach with a median follow-up period of 29.1 months after cCR. During that period no local regrowth or distant metastasis was observed, and the 3-year disease-free and overall survivals were both 100% [22].

### 6.2. Quality of Life Considerations

The choice between surgery and watch-and-wait involves significant quality of life trade-offs that must be carefully considered in the context of dMMR rectal cancer management. Surgery provides definitive treatment with pathological confirmation, but carries risks of permanent functional impairment including bowel dysfunction, sexual and urinary dysfunction, and potential permanent colostomy [23,34,41]. Immunotherapy has shown remarkable success in preserving quality of life for rectal cancer patients achieving CR. Yang et al. found that patients managed with WW after immunotherapy avoided the surgical complications typically associated with traditional treatment, including bowel dysfunction, urinary issues, sexual problems, and ostomy requirements. According to the results, 15.4% of patients received a permanent colostomy and 23.1% a temporary ileostomy [21]. Similarly, Cercek A. et al. demonstrated that complete responders maintained normal bowel, bladder, and sexual function while preserving fertility and reproductive organs in women [13].

Anterior resection syndrome is reported in 30–80% of patients undergoing low anterior resection, while sexual dysfunction from autonomic nerve injury and urinary dysfunction from pelvic nerve damage are also common surgical complications. The avoidance of surgical morbidity through organ preservation provides clear functional advantages. Initial findings indicate improved physical and overall health outcomes with reduced defecatory, sexual, and urinary dysfunction; however, approximately one-third of patients continue to exhibit significant low anterior resection syndrome manifestations [34]. However, some patients may experience improved quality of life with definitive surgical treatment due to reduced anxiety about surveillance requirements and recurrence risk. The psychological benefits of pathological confirmation and treatment completion must be balanced against functional preservation in individual treatment decisions [21].

## 7. Current Clinical Trials and Future Directions

### 7.1. Ongoing Clinical Trials

The AZUR-1 phase II trial represents a critical expansion study aimed at validating the initial 100% CR rate observed with dostarlimab in larger cohorts with extended follow-up periods [47]. The MSK Dostarlimab study is assessing the safety and effectiveness of dostarlimab in patients with locally advanced dMMR rectal cancer, whose response will be then evaluated before considering standard chemoradiation and/or surgery [48]. The Danish Short Course Immunotherapy trial is evaluating the efficacy and tolerability of nivolumab and ipilimumab in patients with stage 1–3 MSI/dMMR rectal cancer [49]. The RESET-C trial is a multicentre, single-arm phase II study assessing neoadjuvant pembrolizumab in stage I–III dMMR colon cancer, with a single dose followed by surgery within 3–5 weeks and follow-up CT scans at 1 and 3 years [50]. The APRAM study represents a randomised, controlled, open-label, multicentre phase III trial specifically designed to evaluate organ preservation in dMMR rectal cancer. It will explore the best anus preservation model for low locally advanced rectal cancer, combining the strategies of consolidation chemotherapy, immunotherapy, and short-course radiotherapy, and aims to preserve the anus of more patients using WW [51].

### 7.2. Future Research Priorities

#### 7.2.1. Treatment Optimisation

Current evidence suggests class effects across pembrolizumab, nivolumab, dostarlimab, and sintilimab, but head-to-head comparisons are needed. The role of combination versus monotherapy approaches requires clarification, with the NICHE studies suggesting potential benefits of dual checkpoint inhibition at the cost of increased toxicity. Establishing optimal treatment duration for sustained responses represents a critical research priority. Current studies employ 6-month treatment courses, but shorter durations may be sufficient given the exceptional response rates observed. Sequencing questions address whether immunotherapy should be used first-line versus in combination with conventional approaches, and the potential role of adjuvant immunotherapy following neoadjuvant treatment.

#### 7.2.2. Novel Therapeutic Development

Next-generation immunotherapy approaches offer potential for enhanced efficacy and broader applicability beyond current checkpoint inhibition strategies. Bispecific antibodies (cadonilimab) targeting PD-1 and CTLA-4 simultaneously may boost anti-tumour activity in patients with dMMR locally advanced rectal cancer [52]. CAR-T cell therapy adapted for solid tumours could provide more potent and sustained immune responses, potentially offering curative approaches for patients with resistant or recurrent disease [53]. Cancer vaccines designed specifically for dMMR tumours may enhance immunological memory and provide long-term protection against recurrence [54].

#### 7.2.3. Biomarker Development and Validation

Tumour mutational burden thresholds are being established to optimise patient selection, with studies suggesting that very high mutational burden (>100 mutations per megabase) correlates with superior responses [4]. Immune infiltration signatures including CD8+ T-cell density, PD-1+ tumour-infiltrating lymphocyte presence, and checkpoint pathway expression levels may provide additional predictive information beyond MSI status. The development of immunoscores to predict immunotherapy response represents an active area of investigation [55]. CtDNA monitoring represents a particularly promising approach for real-time assessment of treatment response and early recurrence detection. Liquid biopsy applications for treatment monitoring may enable more precise assessment of response quality and duration, potentially identifying patients who can safely discontinue treatment or those requiring intensified therapy [30].

## 8. Conclusions

Immunotherapy has revolutionised treatment for dMMR rectal cancer, achieving unprecedented CR rates approaching 100%. This breakthrough challenges traditional surgical paradigms and introduces a novel clinical dilemma: whether patients achieving CR should undergo standard surgery or pursue organ preservation through WW management. The exceptional efficacy has been validated across multiple studies with sustained responses maintaining 100% DFS at extended follow-up. This success creates unprecedented opportunities for organ preservation, particularly important for the typically younger dMMR population where quality of life preservation over decades of life expectancy is paramount.

However, this paradigm shift introduces unique challenges including pseudoprogression and pseudoresidue that require specialised response assessment protocols and multidisciplinary expertise. The choice between surgery and WW represents a decision between two potentially curative approaches, necessitating individualised decision-making that balances organ preservation benefits with patient preferences and compliance capabilities. The remarkable outcomes in dMMR rectal cancer exemplify precision oncology’s potential, demonstrating how molecular subtype-directed therapy can achieve a cure while preserving function. This represents a fundamental shift from anatomical site-based treatment to tumour biology-guided approaches, offering patients the possibility of cure with optimised quality of life.

## Figures and Tables

**Table 1:** Summary of major clinical trials in dMMR colorectal cancer immunotherapy.

	Phase	Treatment	N	Setting	CR Rate (%)	Grade 3–4 AE (%)	Median Follow-Up (mo)	Recurrence (%)
KEYNOTE-177 [8]	III	Pembrolizumab	153	MCRC	13.1 rCR	22	44.5	NR
CheckMate-142 [7]	II	Nivolumab Nivolumab + Ipilimumab	74 45	MCRC	2.7 rCR 13 rCR	20 6	12 29	NR
NICHE-1 [10] NICHE-2 [11] NICHE-3 [12]	II	Nivolumab + Ipilimumab Nivolumab + relatlimab	32 111 59	LACC	69 pCR 68 pCR 68 pCR	12 4 10	25 26 8	0 0 2
Cercek A., et al., 2022 [13] 2024 [16]	II II	Dostarlimab + WW	12 41	LARC	NR 100 cCR	0	12 28.9	0
PICC [17]	II	Toralimab + celecoxib Toralimab	17 17	LACRC	88 65	3	14.9	0
Gögenur I., et al. [18]	II	Pembrolizumab	42	LACC	46 pCR	7.1	NR	NR
Chen G., et al. [19]	II	nIT + surgery nIT + WW	6 9	LARC	75 pCR + cCR	6	17.2	0
Zhang X., et al. [20]	Retro	nIT + surgery nIT + WW	29 3	LACRC	75.9 pCR	0	14	0 0
Yang R., et al. [21]	Retro	nIT + surgery nIT + WW	13 7	LARC	84.6 pCR	0	25	0 0
Yu J., et al. [22]	Retro	nIT + WW	24	LARC	100 cCR	8.4	29.1	0
Wang Q.X., et al. [23]	Retro	nIT + surgery nIT + WW	10 19	LARC	pCR NR	15	17.1	0
Xie Y., et al. [24]	Retro	nIT + surgery nIT + WW	12 1	LARC	92 pCR	NR	NR	NR

**Table 3:** Surveillance Protocol for Watch-and-Wait Management Following Neoadjuvant Immunotherapy [NCCN Guidelines Version 3.2025].

Time Period	History and Physical Examination	CEA	DRE and Proctoscopy or FS	MRI Pelvis	CT CAP
0–24 months	Every 3–6 months	Every 3–6 months	Every 3–4 months	Every 6 months	Every 6–12 months
24–60 months	Every 6 months	Every 6 months	Every 6 months	Every 6 months (until the 36th month)	Every 6–12 months

## Abbreviations

The following abbreviations are used in this manuscript:

TME	Total mesorectal excision
dMMR	Deficient mismatch repair
MSI-H	High microsatellite instability
ICI	Immune checkpoint inhibitors
CR	Complete response
pCR	Pathological complete response
cCR	Clinical complete response
rCT	Radiological complete response
AE	Adverse events
Retro	Retrospective
NR	Not reported
MCRC	Metastatic colorectal cancer
LACRC	Locally advanced colorectal cancer
LACC	Locally advanced colon cancer
LARC	Locally advanced rectal cancer
OR	Objective response
PFS	Progression free survival
DFS	Disease free survival
DRE	Digital rectal examination
WW	Watch-and-wait
NCCN	National comprehensive cancer network
ESMO	European society of medical oncology
ASCO	American society of clinical oncology
ctDNA	Circulating tumour DNA
nIT	Neoadjuvant immunotherapy
MSK	Memorial Sloan Kettering
FS	Flexible sigmoidoscopy
CT CAP	Computed tomography chest-abdomen-pelvis
MRI	Magnetic resonance imaging
PET	Positron emission tomography
FDG	Fluorodeoxyglucose
mrTRG	Magnetic resonance tumour response grade
RECIST	Response evaluation criteria in solid tumours
PD	Progressive disease
iUPD	Unconfirmed progressive disease
iCPD	Confirmed progressive disease
AJCC	American Joint Committee on Cancer
